# PERITUMORAL BUDDING AS A PREDICTOR FOR LYMPH NODE METASTASES IN COLORECTAL CARCINOMAS: WHAT IS THE IMPORTANCE?

**DOI:** 10.1590/0102-6720202500006e1875

**Published:** 2025-04-07

**Authors:** Emily Karoline Araujo Nonato dos SANTOS, Bruna Gama TRICHES, Guilherme Prestes da SILVA, Julia Costa LINHARES, Samya Hamad MEHANNA, Marcela Santos CAVALCANTI

**Affiliations:** 1 Faculdade Evangélica Mackenzie do Paraná, Medical Course - Curitiba (PR), Brazil.; 2 Faculdade Pequeno Príncipe, Medical Course - Curitiba (PR), Brazil.; 3 Faculdade Evangélica Mackenzie do Paraná, Department of Pathology - Curitiba (PR), Brazil

**Keywords:** Colorectal Neoplasms, Lymphatic Metastasis, Prognosis, Neoplasias Colorretais, Metástase Linfática, Prognóstico

## Abstract

**BACKGROUND:**

Microscopic analysis of tumor budding (TB) may be an essential predictive tool for regional lymph node metastases in colorectal cancer, especially among patients in intermediate stages, who exhibit considerable prognostic variability.

**AIMS:**

The aim of this study was to assess the predictive power of BT regarding the presence of lymph node metastases and its association with other characteristics related to colorectal carcinoma progression.

**METHODS:**

This is a cross-sectional, retrospective study with a quantitative approach, focusing on the review of medical records and histopathological reports of patients who underwent oncologic surgery for colorectal cancer.

**RESULTS:**

A total of 153 patient records were examined, with a predominance of the 61-70 age group and a male majority (50.98%). Adenocarcinoma not otherwise specified was the most common histological type (60.78%), with the majority exhibiting moderate differentiation (87.58%). From the total sample, 97 cases (63.39%) exhibited TB, with 51.55% classified as a high budding score. Invasion of adipose tissue/subserosa was the most prevalent, occurring in 46.41% of cases. Regional lymph node metastases and angiolymphatic invasion were observed in 66 and 101 patients, respectively. Cross-tabulation analysis showed a statistically significant association between TB and lymph node metastasis (p<0.05).

**CONCLUSIONS:**

The relationship between TB and lymph node metastasis highlights the significance of this histological factor in the risk stratification and prognosis of patients with colorectal cancer, complementing TNM staging. Therefore, the assessment of tumor budding is crucial in histopathological reports, potentially influencing additional therapeutic decisions.

## RESUMO

**RACIONAL:**

A análise microscópica de brotamento peri-tumoral (BT) pode ser uma importante ferramenta preditiva de metástases linfonodais regionais em casos de câncer colorretal, principalmente entre pacientes em estágios intermediários, os quais apresentam marcante variabilidade prognóstica.

**OBJETIVOS:**

Avaliar o poder preditivo do BT em relação à presença de metástases linfonodais, bem como sua associação com outras características relacionadas à progressão do carcinoma colorretal.

**MÉTODOS:**

Trata-se de estudo transversal, retrospectivo com abordagem quantitativa, com revisão de prontuários médicos e laudos anatomopatológicos de pacientes submetidos a cirurgia oncológica por câncer colorretal.

**RESULTADOS:**

Foram analisados 153 prontuários de pacientes, predominando a faixa etária de 61-70 anos, com uma maioria masculina (50,98%). O adenocarcinoma sem especificação foi o tipo histológico mais comum (60,78%), com a maioria mostrando moderada diferenciação (87,58%). Da amostra total, 97 casos (63,39%) apresentaram BT, com 51,55% classificados como alto escore de brotamento. A invasão em tecido adiposo/subserosa foi a mais prevalente, ocorrendo em 46,41% dos casos. Metástases em linfonodos regionais e embolização angiolinfática foram observadas em 66 e 101 pacientes, respectivamente. A análise cruzada indicou uma associação estatisticamente significativa entre BT e metástase linfonodal (p<0,05).

**CONCLUSÕES:**

A relação entre BT e metástase linfonodal destaca a importância deste fator histológico na estratificação de risco e prognóstico em pacientes com câncer colorretal, complementando o estadiamento TNM. Portanto, a avaliação do brotamento tumoral é crucial em laudos anatomopatológicos, podendo influenciar decisões terapêuticas adicionais.

**Figure:**
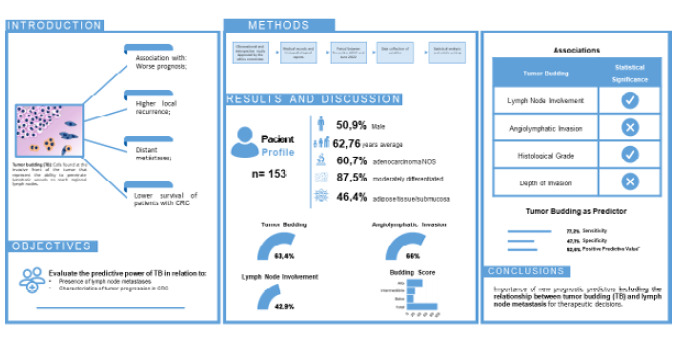
Peritumoral budding as a predictor for lymph node metastases in colorectal carcinomas: what is its significance?

**Central Message**

Colorectal cancer (CRC) is the second-most prevalent cancer in both sexes in Brazil, excluding non-melanoma skin cancer, and is associated with significant morbidity and mortality. The prognosis of CRC is based on the disease stage according to the TNM classification. However, there is considerable variability in outcomes and prognosis among patients at identical stages, especially in intermediate stages (II and III). Thus, additional strategies for stratifying the risk of recurrence and metastasis, designed to enhance the treatment of CRC patients, present a favorable alternative by utilizing the tumor budding (TB) marker.

**Perspectives**

This study highlights the significant relationship between the presence of TB and lymph node metastasis, emphasizing the importance of tumor budding as a detectable histological prognostic factor in patients with colorectal cancer. TNM staging remains the primary stratification method; however, assessing other prognostic predictors in CRC patients is crucial for oncological treatment decisions.

## INTRODUCTION

Colorectal cancer (CRC) is the second-most prevalent cancer in both sexes in Brazil, excluding non-melanoma skin cancer, and is associated with significant morbidity and mortality^11^. The prognosis of CRC is largely based on the disease stage according to the TNM classification^12^. However, there is significant variability in outcomes and prognosis among patients at identical stages, especially in intermediate stages (II and III)^16^. Therefore, additional strategies to stratify the risk of recurrence and metastasis, aiming to enhance the treatment of CRC patients, may provide beneficial alternatives through the utilization of the tumor budding (TB) marker^14^.

TB is a phenomenon where single isolated cells or small clusters of up to four cells are present at the invasive front of the tumor, indicating the tumor’s capacity to infiltrate the extracellular matrix and lymphatic vessels to reach regional lymph nodes^19^. This process has been associated with worse prognosis, including higher local recurrence, distant metastases, and reduced survival in CRC patients^16^. In 2016, the International Tumor Budding Consensus Conference (ITBCC) standardized TB assessment in CRC patients into three distinct groups based on the number of buds identified under microscopy:

1. Low TB group: a maximum of four buds in the assessment field (hotspot area);
2. Intermediate TB group: between five and nine buds;
3. High TB group: 10 or more buds^8^.

The aim of this study was to assess the predictive power of TB regarding the presence of lymph node metastases, and other characteristics of tumor progression in CRC, in a Brazilian university hospital.

## METHODS

This is an observational, retrospective study with a quantitative approach, conducted in Curitiba (PR), Brazil. Medical records from the Mackenzie Evangelical University Hospital (HUEM) were examined, along with histopathological results of resected surgical specimens from patients who underwent oncological surgery for CRC between November 2019 and June 2022. Patients with missing data in medical records and/or incomplete histopathological analyses were excluded. Multiple variables, such as sex, age, type/grade of histological neoplasia, depth of invasion, angiolymphatic embolization, lymph node metastases, and TB assessment, were considered.

For analysis, the data were exported to a Microsoft Office Excel spreadsheet, where descriptive statistics were used, including absolute numbers and percentages. The chi-squared test was performed when applicable with a significance level of <0.05. The study was approved by the HUEM Research Ethics Committee (nº 70071623.7.0000.0103), in accordance with Resolution no. 466/12 of the National Research Council, which regulates scientific research in human subjects. The data obtained were compared with the literature on the subject.

## RESULTS

**Figure 1:**
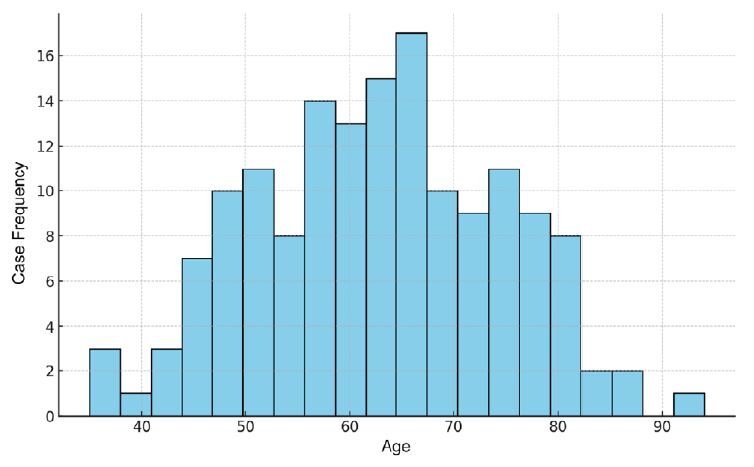
Distribution of patients with colorectal cancer by age groups (n=153).

From November 2019 to June 2022, 153 CRC cases were recorded at the hospital’s high-complexity center that met the inclusion criteria for this study. The distribution between sexes was similar, with a slight predominance of males, accounting for 50.98% of the cases. Age ranged from 35 to 94 years, as shown in Figure 1, with a mean of 62.76±12.02 and with 30.07% predominantly in the age group of 61-70 years. Figure 1 shows the distribution of CRC patients by age group (n=153).

Adenocarcinoma not otherwise specified (NOS) was the most prevalent histological type, present in 93 patients (60.78%) of the cases. Table 1 shows the degree of tumor differentiation, with the majority classified as moderately differentiated, totaling 134 cases (87.58%).

**Table 1:** Distribution of colorectal cancer differentiation degree (n=153).

Differentiation degree	n	%
Moderately differentiated	134	87.58
Poorly differentiated	16	10.45
Well-differentiated	3	1.96
Total	153	100

Table 2 shows that in 66 patients (43.13%), at least one metastasis in regional lymph nodes was detected in the surgical specimen.

**Table 2:** Distribution of colorectal cancer patients according to lymph node involvement (n=153).

Lymph node involvement	n	%
Not involved	87	57.14
Involved	66	42.86
Total	153	100

The analysis of tumor invasion depth (pathological staging T) of the samples showed a significant number of cases classified as pT3 (46.41%), characterized by tumor cell invasion into the pericolic adipose tissue, followed by pT4a (30.07%), which includes infiltration to the serosal surface. Additionally, the presence of pT2 (16.69%), characterized by the infiltration of the muscularis propria, was also particularly noteworthy. The relationship between tumor depth and lymph node metastasis presence was analyzed and presented in Table 3. There was a statistically significant association between tumor invasiveness and the probability of lymph node involvement (p<0.05).

**Table 3:** Tissue invasion depth (T) in colorectal cancer (n=153).

Tissue infiltration	pT	n	%	Lymph node metastasis (n)	p-value
Mucosa	pTis	1	0.65	0	
Submucosa	pT1	4	2.6	0	
Muscularis propria	pT2	24	16.69	3	
Adipose tissue	pT3	71	46.41	33	
Serosa	pT4a	46	30.07	27	
Adjacent organs	pT4b	7	4.58	3	
Total	-	153	100	66	0.0032

Angiolymphatic embolization was examined and found in 101 patients (66.01%). However, in 53 cases (34.64%), it was not identified.

TB identification was described in 97 cases (63.39%), classified into low, intermediate, and high scores, as shown in Table 4. Over half of these cases had the highest score.

**Table 4:** Distribution of budding score according to classification into high, intermediate, or low (n=97).

Budding score	n	%
High	50	51.55
Intermediate	26	26.80
Low	21	21.65
Total	97	100

**Figure 2:**
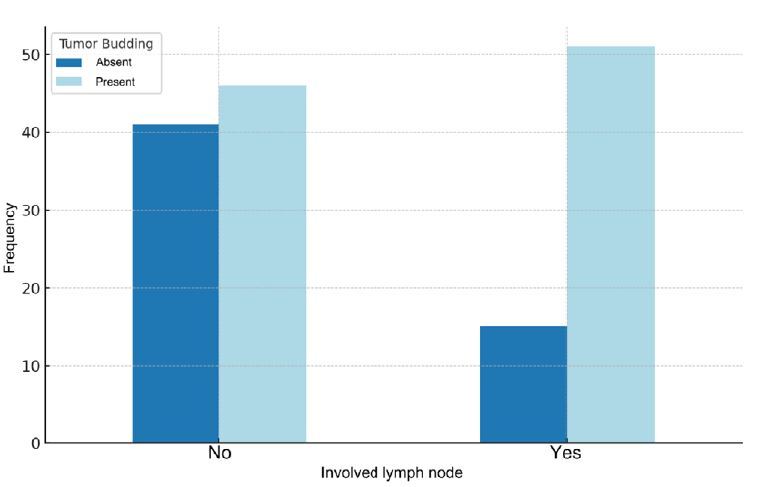
Relationship between lymph node involvement and tumor budding.

ROC: receiver operating characteristic; AUC: area under the curve.

The association between TB and lymph node metastasis was considered significant (p<0.05) since 51 of the 66 patients with lymph node metastasis had TB. Conversely, most patients without lymph node involvement had positive TB (Figure 2). Furthermore, the sensitivity and specificity of peritumoral budding for regional lymph node involvement were 77.27% and 47.13%, respectively, with a positive predictive value of 52.58%.

**Figure 3:**
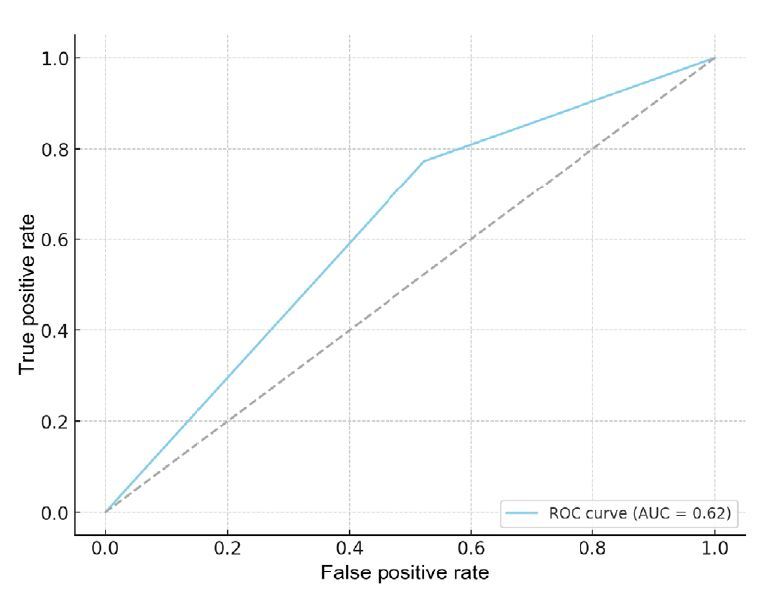
The blue curve shows the test performance; the dashed gray line shows a test with no discriminative ability. The area under the curve of 0.622 indicates moderate test accuracy.

Additionally, the ROC (receiver operating characteristic) curve analysis for TB on lymph node involvement in CRC patients revealed an area under the curve (AUC) of 0.622, as shown in Figure 3. This value indicates moderate accuracy of TB in distinguishing between cases with and without lymph node involvement. Moreover, likelihood ratios were calculated, with a positive likelihood ratio of approximately 1.46 suggesting that a positive TB test result increases the probability of lymph node involvement by approximately 46%. In contrast, a negative likelihood ratio of approximately 0.46 indicates that a negative result reduces the probability of lymph node involvement by approximately 46%.

Finally, TB was associated with lymph node metastasis, histological grade, invasion depth, and angiolymphatic embolization. However, the latter exhibited no statistical significance, as shown in Table 5.

**Table 5:** Age, sex, lymph node involvement, angiolymphatic invasion, histological grade, and invasion depth in CRC patients with and without tumor budding (n=153).

	Tumor budding		p-value
	Present (n)	Absent (n)	
	97	56	
Age (mean)	62.65	62.86	0.877*
Sex (n)			
Male	52	27	
Female	45	30	
Lymph node metastasis (n)			
Present	51	15	0.0034^†^
Absent	46	41	
Angiolymphatic invasion (n)			
Present	68	33	0.2192^†^
Absent	29	23	
Histological grade (n)			
Well-differentiated	0	3	0.0131^†^
Moderately differentiated	90	44	
Poorly differentiated	7	9	
Invasion depth			
Mucosa	0	1	0.068^†^
Submucosa	0	4	
Muscularis propria	15	9	
Adipose tissue	45	26	
Serosa	32	14	
Adjacent organs	5	2	

*Student’s t-test; ^†^χ2 test.

## DISCUSSION

Most of the 153 patients with CRC surgically treated in a high-complexity center were male (50.98%). These findings align with current data from the World Health Organization (WHO) for Brazil, where CRC exhibits an almost equal distribution between sexes, with males accounting for 49.6% of the total CRC cases^6^ ^,^ ^18^. The American Cancer Society (ACS) estimates that in 2022, there were 80,690 new cases among males, compared to 70,340 new cases among females^1^.

The predominant age group in this study was between 61 and 70 years, with a mean age of 62 years. Age assessment is crucial as it is a risk factor for CRC development, with cases being more common in patients over 50 years and significantly increasing with each decade. The average age at diagnosis is 72 years for women and 68 years for men^10^. A Brazilian study of 521 cases found an average age of 63 years, with an affected range between the fifth and the eighth decades of life^3^.

The most widely used and gold-standard pathological staging system for CRC evaluation is the TNM system, recommended by the American Joint Committee on Cancer (AJCC), which considers tumor invasion depth (T); the number of compromised lymph nodes (N); and the presence or absence of distant metastases (M)^12^ ^,^ ^20^. Our study showed a prevalence of 71 (46.41%) cases with infiltration into the pericolic adipose tissue, corresponding to pT3, which is also described as predominant in the global literature^3^ ^,^ ^4^ ^,^ ^17^. Additionally, at least one lymph node metastasis was identified in 66 patients (42.86%). A similar value has been described in the literature^13^.

TB was identified in 97 (63.39%) cases, with the majority classified as high scores, corresponding to 51.55% of the results. Furthermore, a statistically significant association was found between peritumoral budding and the presence of lymph node metastasis (p<0.05), with 77.27% sensitivity.

Although the mechanism of TB formation in colorectal cancer is not yet fully understood, the predominant theory suggests that at least some types of budding represent an example of epithelial-mesenchymal transition. It suggests that during the epithelial-mesenchymal transition, tumor cells undergo changes that include the loss of cell adhesion molecules, cytoskeletal modifications, increased production of extracellular matrix components, resistance to apoptosis, and the ability to degrade the basement membrane^7^.

These described changes result in a cellular phenotype with greater migratory and invasive capacity, allowing tumor cells to detach from the primary tumor mass and disseminate to adjacent tissues, lymph nodes, and distant organs^7^. Therefore, TB is considered an early step in the neoplasm’s ability to invade lymphovascular spaces and metastasize. Additionally, TB can currently be used as an auxiliary in CRC risk stratification^2^ ^,^ ^15^ ^,^ ^19^.

In 46 (30.06%) patients, the presence of TB was detected without lymph node involvement; yet, this finding suggests increased tumor aggressiveness, being associated with infiltration depth and substantial loss of cellular differentiation. Histopathological detection is crucial in assessing patients with early stage CRC due to its association with the presence of lymph node metastases, indicating the need for additional resections or association with other complementary therapeutic approaches, such as adjuvant oncological therapy, and in metastatic cases, it is associated with a worse prognosis^5^ ^,^ ^15^.

Marx et al. have shown a statistically significant association between TB and lymphovascular invasion^9^. However, in this study, this association was not confirmed. Several factors may justify this outcome, including imprecise histological assessment, technical difficulties in histotechnical processing, insufficient macroscopic sampling, or the fact that these were initial cases and TB had not yet resulted in subsequent embolization.

The ITBCC recommends the assessment of TB using hematoxylin and eosin-stained histological slides, and a hotspot at the invasive tumor front (standard field size of 0.785 mm^2^). To assist this analysis, the consensus provides a table for standardizing this count across various microscopes^8^. According to ITBCC, TB should be included in CRC protocols and described in patients’ pathology reports due to its prognostic, clinical, and therapeutic value. Thus, this histopathological evaluation should always be described. In the present study, information was sourced from radical surgical specimens; nonetheless, TB identification is particularly important in endoscopic resections of early cases, such as polypectomies and mucosectomies^8^.

In recent years, there has been a growing increase in CRC diagnosis, with local resection surgery considered curative in the absence of metastases^5^. TNM classification remains the primary staging stratification method. However, evaluating other new prognostic predictors in CRC patients is uniquely important in therapeutic decision-making in oncology.

## CONCLUSIONS

This study established a significant relationship between the presence of TB and lymph node metastasis, emphasizing the relevance of tumor budding as a detectable histological prognostic factor in patients with colorectal cancer.
